# Reply: Management and Imaging Strategies in Pediatric Infective Endocarditis With Mitral Prolapse

**DOI:** 10.1016/j.jaccas.2025.106418

**Published:** 2026-01-07

**Authors:** Monica Barki, Adelaide Iervolino, Giacomo Ingallina, Francesco Ancona, Eustachio Agricola

**Affiliations:** a IRCCS San Raffaele Hospital, Milan, Italy; b Federico II University Hospital, Naples, Italy

We thank the colleagues for their thoughtful and helpful comments on our case report.^1^ We appreciate the opportunity to clarify several pivotal points raised in their letter.

First, regarding imaging, in our case, 3-dimensional multiplanar reconstruction was performed and 3-dimensional imaging of the mitral valve en face view is displayed in Figure 2. This technique was helpful in identifying the new lesion with high precision and in confirming the new P3 origin of the regurgitation. We agree that advanced imaging improves diagnostic yield, and we routinely use it in our clinical practice, particularly in suspected cases of infective endocarditis (IE).

Second, concerning management strategy, the case cited by the colleagues involved mitral valve endocarditis with significant vegetations necessitating transcatheter aspiration before intervention. In our case, no vegetation was present and the diagnosis was based on the presence of a new regurgitation and the positivity at blood cultures.^2^ As for the study presented, the children treated surgically were patients affected by mitral valve disease rather than mitral valve endocarditis.^3^ This clinical distinction is crucial because perioperative mortality in active IE is substantially higher than in noninfective mitral pathology. Current guidelines recommend surgical intervention in IE when there is severe valvular regurgitation causing heart failure, uncontrolled infection, or high embolic risk from large vegetations.^4^ None of these criteria were met in our patient. She remained asymptomatic, without extravalvular involvement and with preserved pulmonary pressures even on stress echocardiography.^5^

Importantly, surgery in the acute infective phase carries increased perioperative mortality, particularly in mitral valve IE. A recent meta-analysis demonstrated that early surgery after neurologic complications was associated with nearly doubled operative mortality compared with delayed intervention.^6^ Additionally, observational registries confirm that mitral valve surgery in the setting of IE remains associated with higher short-term mortality.^7^

Finally, regarding risk assessment, although pediatric tools such as Pediatric Risk of Mortality scores have prognostic utility in critical care, our patient was clinically stable with no significant systemic complications; therefore, such tools would not have influenced management.

In summary, our decision to pursue conservative strategy reflects a tailored, evidence-based care in a 14-year-old girl with IE complicating mitral valve prolapse. Surgery was deferred in view of the absence of complications, the high risk of operating during active infection, and benefits of postponing intervention until adulthood when valve repair and prosthetic options are more durable.

## References

[1] Iervolino A., Barki M., Ingallina G. (2025). New mitral regurgitation unveiling infective endocarditis in an adolescent with mitral valve prolapse. JACC Case Rep.

[2] Rajagopal D.R., Jayanthi R., Bolivar Aldana J.J. (2025). Transcatheter treatment of patient with mitral valve endocarditis and severe aortic stenosis and insufficiency. JACC Case Rep.

[3] Sicim H., Alaeddine M., Velez D.A. (2025). Single center experience with surgically implanted Melody and Sapien 3 valves in the mitral position in young children. Front Cardiovasc Med.

[4] Kovac J. (2023). 2023 ESC guidelines for the management of endocarditis developed by the task force on the management of endocarditis of the European Society of Cardiology (ESC) endorsed by the European Association for Cardio-Thoracic Surgery (EACTS) and the European Association of Nuclear Medicine (EANM). Eur Heart J.

[5] Praz F., Borger M.A., Lanz J. (2025). 2025 ESC/EACTS Guidelines for the management of valvular heart disease. Eur Heart J.

[6] Tam D.Y., Yanagawa B., Verma S. (2018). Early vs late surgery for patients with endocarditis and neurological injury: a systematic review and meta-analysis. Can J Cardiol.

[7] Østergaard L., Smerup M.H., Iversen K. (2020). Differences in mortality in patients undergoing surgery for infective endocarditis according to age and valvular surgery. BMC Infect Dis.

